# Thoracic paravertebral block reduced the incidence of chronic postoperative pain for more than 1 year after breast cancer surgery

**DOI:** 10.1186/s40981-015-0023-4

**Published:** 2015-10-26

**Authors:** Hiroki Shimizu, Yoshinori Kamiya, Hironobu Nishimaki, Sadahei Denda, Hiroshi Baba

**Affiliations:** 1 Division of Anesthesiology, Niigata University Graduate School of Medical and Dental Sciences, 1-757 Asahimachi-dori, Chuo Ward, Niigata, Niigata 951-8510 Japan; 2 Department of Anesthesiology, Niigata City General Hospital, 463-7 Shumoku, Chuo Ward, Niigata, Niigata 950-1197 Japan; 3 Department of Anesthesiology, Uonuma Institute of Community Medicine, Niigata University Medical and Dental Hospital, 4132 Urasa, Minami-uonuma, Niigata 949-7302 Japan

**Keywords:** Chronic postoperative pain, Thoracic paravertebral block, Breast cancer surgery

## Abstract

**Background:**

Thoracic paravertebral block (TPVB) is used to reduce pain after breast cancer surgery (BCS), but the pain-reduction effects more than 1 year after surgery are unclear.

**Findings:**

Fifty-one patients underwent BCS at the Niigata City General Hospital from December 2009 through March 2010. To evaluate the long-term effects of TPVB in the reduction of chronic pain after BCS, we retrospectively reviewed the anesthesia charts and medical records of these patients and conducted telephone surveys regarding postoperative pain 13–17 months after surgery in 46 of these patients. Among the 46 patients enrolled in this study, 17 experienced chronic pain. There was a significant difference in the percentage of patients that received TPVB among those with and without chronic pain (patients with chronic pain 5/17 (29.4 %), patients without chronic pain 18/29 (62.1 %), *p* = 0.039). The pain score 3–6 h after surgery was significantly higher in the patients with chronic pain than without (*p* = 0.016). Bivariate logistic regression revealed that TPVB and pain score 3–6 h after surgery were independent predictive factors of chronic pain after BCS.

**Conclusions:**

These results indicate that TPVB has the potential to reduce chronic pain for more than 1 year after BCS.

## Findings

### Introduction

Breast cancer is the most commonly diagnosed malignancy in women, and the incidence of this condition is increasing [1, 2]. Because of the desirability of breast-conserving surgical techniques and the use of sentinel lymph node sampling, less invasive breast cancer surgery (BCS) procedures have been developed [3–5]. However, after BCS, up to 80 % of patients experience chronic postoperative pain [6, 7]. The exact cause of chronic postoperative pain after BCS remains unclear, but there is an association between acute pain and chronic pain generation after BCS [8–11].

Thoracic paravertebral block (TPVB) is a commonly used adjuvant analgesic technique in BCS, and previous study reported that TPVB reduces intraoperative analgesic requirements and suppresses acute postoperative pain [12, 13]. However, the influence of TPVB on chronic postoperative pain after BCS remains controversial [14–16].

We hypothesized that TPVB combined with general anesthesia may reduce acute and chronic postoperative pain after BCS. We analyzed the association between the incidence of chronic postoperative pain after BCS and the use of TPVB combined with general anesthesia by using a retrospective chart review and telephone survey.

### Case series

#### Study subjects

This retrospective cohort study was performed at Niigata City General Hospital. This study was reviewed and approved by the Niigata City General Hospital institutional review board (approval number 14–047). Written informed consent was obtained from the patient, and we confirmed the consent for publication of this case series when we performed telephone survey to the patients. We reviewed the anesthesia charts and medical records of all patients who underwent BCS under general anesthesia with or without TPVB from December 2009 through March 2010. Patients who underwent bilateral BCS or simultaneous BCS and surgery involving another part of the body were excluded. The same anesthesiologist (HS) performed TPVB with general anesthesia in all patients. The director of the division (SD) assigned the anesthesia cases of the day to an individual anesthesiologist, and certain types of cases were not assigned to a specific individual.

#### Analgesia and data collection

General anesthesia was induced with propofol and remifentanil and maintained with propofol or sevoflurane and remifentanil. The propofol infusion rate was regulated by target-controlled infusion (target blood concentration 1.8–4.0 μg/ml with Terumo TE372 TCI [Diprifusor]). The patients were monitored using an electrocardiogram, non-invasive arterial blood pressure (every 5 min), pulse oximeter, and bispectral index^™^ (BIS^™^) monitor (Aspect Medical Systems, Leiden, The Netherlands). Target BIS values were set between 40 and 60. We adjusted the remifentanil dose to maintain systolic blood pressure of 80–140 mmHg, heart rate of 50–100 beats/min, and BIS values of 40–60. TPVB was performed under ultrasound guidance before induction of general anesthesia by administering 20-ml 0.375 or 0.5 % ropivacaine in one or two intercostal spaces between T2/3 and T4/5. Non-TPVB patients received fentanyl and flurbiprofen axetil as transitional analgesia.

The medical records were reviewed by an anesthesiologist (HN) blinded to the anesthesia method to determine the intensity of postoperative pain at 0–3, 3–6, and 6–24 h after surgery according to a four-point verbal rating scale (0: none, 1: mild pain, 2: moderate pain, 3: severe pain). Flurbiprofen axetil, loxoprofen, and acetaminophen were used as rescue analgesics.

An anesthesiologist (HN) interviewed the patients by phone to determine the postoperative pain 13–17 months after surgery. Chronic pain was defined as pain in the surgical area or the ipsilateral arm, present at least 4 days a week, with an intensity of one or more on the four-point verbal rating scale, described as a typical neuropathic pain consisting of burning pain, shooting pain, pain evoked by pressure, and deep blunt pain [17].

We recorded patient age, height, weight, body mass index (BMI), surgical procedure, intraoperative average remifentanil dose and analgesia (with or without TPVB), postoperative acute pain within 24 h, postoperative adjuvant chemotherapy and radiotherapy, duration from surgery to telephone survey, and cancer recurrence. We evaluated which parameters were correlated with chronic postoperative pain.

#### Data analyses

Data for continuous variables are presented as the mean ± SD; data for categorical or ordinal variables, and data that did not conform to a normal distribution, are presented as the median [range]. Demographic data were analyzed by paired *t* test or one-way analysis of variance (ANOVA). Univariate analyses were performed using the Mann–Whitney *U* test or Fisher’s exact test. All statistical analyses were performed using Microsoft Excel 2011 for Macintosh (Microsoft, Redmond, WA, USA) with a statistical macro (XLSTAT2014; Addinsoft, New York, NY, USA). We considered *p* < 0.05 statistically significant.

### Results

**Fig. 1:**
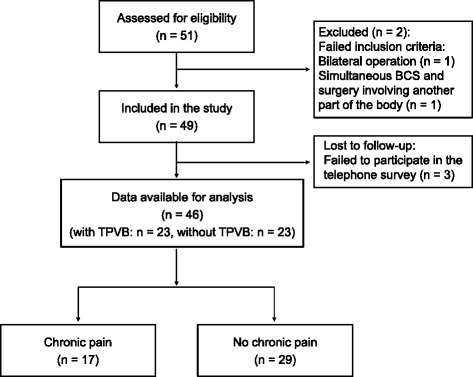
CONSORT diagram of this study

Fifty-one patients underwent BCS during the study period. After exclusion of patients who underwent bilateral breast surgery or simultaneous surgery involving another body part, we identified 49 patients as a telephone survey conducted in May 2011. The telephone survey was conducted in May 2011 and 46 patients answered the survey completely (Fig. 1).

**Fig. 2:**
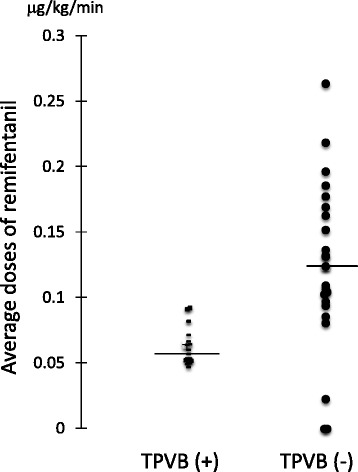
Average doses of remifentanil administered during breast cancer surgery in patients with or without TPVB. In patients with TPVB, the remifentanil doses were significantly smaller than in those without TPVB

We used 0.375 % ropivacaine for 3 cases and 0.5 % ropivacaine for 17 cases. There was no difference in the average dose of remifentanil during surgery (0.375 % 0.068 ± 0.011 μg/kg/min vs. 0.5 % 0.064 ± 0.016 μg/kg/min). Therefore, we pooled the data for patients treated with the two concentrations of ropivacaine. The average dose of remifentanil was significantly lower in the patients with TPVB than in those without (0.064 ± 0.016 μg/kg/min vs. 0.124 ± 0.064 μg/kg/min, *p* < 0.0001; Fig. 2).

**Fig. 3:**
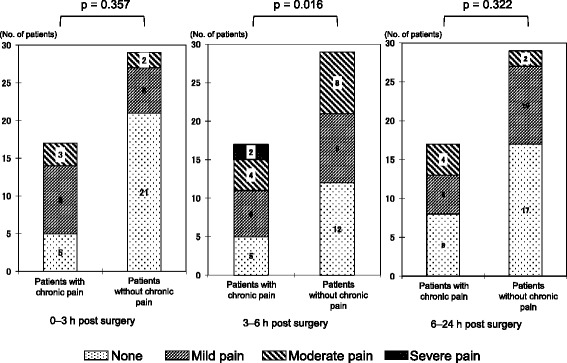
Patient distribution of postoperative pain scores 0–3, 3–6, and 6–24 h after surgery in patients with and without chronic pain. The pain score 3–6 h after surgery was significantly higher in the patients with chronic pain than in the patients without chronic pain. However, there was no significant difference in the pain scores 0–3 and 6–24 h after surgery between the two groups, although the patients with chronic pain tended to experience more pain than the patients without chronic pain at each time interval

The interval between surgery and the telephone survey ranged from 13 to 17 months. Seventeen of 46 (37 %) patients suffered chronic postoperative pain. We compared the demographic data, anesthetic method, postoperative pain score, and perioperative adjuvant cancer therapy between patients with and without chronic pain (Table 1). There were no significant differences in patient characteristics, operative procedure, duration since the operation, postoperative acute pain within 24 h after surgery, and the incidence of required rescue analgesics between the patients with and without chronic pain. The percentage of patients that received TPVB was significantly smaller among those with chronic pain than among those without (5/17 (29.4 %) vs. 18/29 (62.1 %), *p* = 0.039). However, there was no difference in the worst pain score within 24 h of surgery between the two groups (*p* = 0.33). The pain scores 3–6 h after surgery were significantly higher for those with chronic pain than those without. The pain scores 0–3 and 6–24 h after surgery were not significantly different between the two groups (Fig. 3). On the other hand, we compared 23 patients who received TPVB with the other 23 patients who did not receive TPVB about postoperative pain score. The pain scores recorded during the first 24 h after surgery tended to be lower for those who received TPVB than for those who did not (0–3 h; *p* = 0.076, 3–6 h; *p* = 0.056, 6–24 h; *p* = 0.048). Moreover, incidence of chronic pain was significantly lower in the patients who received TPVB than the patients who did not received (21.7 % [5/23] vs. 52.2 % [12/23]).

**Table 1:** Demographic data and postoperative outcomes

	Chronic pain (*n* = 17)	No chronic pain (*n* = 29)	*p* value
Age (years)	55.7 ± 11.7	57.4 ± 12.4	0.78
Height (cm)	156.2 ± 6.9	155.3 ± 6.0	0.60
Body weight (kg)	56.8 ± 10.9	55.7 ± 9.1	0.82
Body mass index	23.3 ± 4.0	23.1 ± 3.1	0.97
Type of surgery			
Lumpectomy	10	13	0.38
Mastectomy	7	16	
No. of axillary lymph nodes removed	4	5	0.71
Treatment received			
Surgery alone	16	26	
Surgery + radiation	0	1	1
Surgery + chemotherapy	0	1	
Surgery + radiation + chemotherapy	1	1	
Postsurgery (months)	15.2 ± 1.4	14.7 ± 1.3	0.18
No. of perioperative thoracic paravertebral nerve blocks	5	18	0.039
Postsurgery acute pain (<24 h)			
Severe	2 (11.8 %)	0 (0 %)	
Moderate	6 (35.3 %)	9 (31.0 %)	0.33
Mild	5 (29.4 %)	11 (37.9 %)	
None	4 (23.5 %)	9 (31.0 %)	
Incidence of rescue analgesics	1 [0–4]	0 [0–3]	0.23
No. of cancer recurrence	0	0	1

Data are shown as mean ± SD or median [range]

## Discussion

Study results revealed that TPVB combined with general anesthesia reduced the occurrence of postoperative chronic pain for more than 1 year after BCS and that postoperative acute pain 3–6 h after surgery was a positive predictive factor for chronic postoperative pain.

We defined chronic postoperative pain as intermittent pain more than 4 days in a week [17], and the incidence (37 %) was in accordance with previous reports [6, 7]. Compared with general anesthesia alone, TPVB combined with general anesthesia enhances recovery after surgery [12, 13]. However, there are few reports on the effects of TPVB on chronic pain after mastectomy [14, 15]. In one study, TPVB with general anesthesia reduced the severity of chronic pain, although it failed to reduce the relative risk of chronic pain, up to 6 months after modified radical mastectomy [16]. In our study, TPVB reduced postoperative chronic pain for more than 1 year after surgery, as previously reported [15].

Mechanisms whereby TPVB prevents chronic pain after BCS remain unknown, but cumulative evidence suggests that the intensity of acute postoperative pain is associated with the incidence of chronic pain [8–11, 18]. In this survey, the pain score 3–6 h after surgery was significantly less in patients without chronic pain than in those with chronic pain. In previous studies, preoperative TPVB in BCS reduced the postoperative pain scores and the requirement for supplemental analgesics for at least 24 h after surgery [12, 13]. Moreover, regional anesthesia preceding surgery reduced the progression of postoperative pain and the requirement for supplemental analgesia [19], which is consistent with our findings.

This study has certain limitations. First, it is retrospective, and a prospective randomized study is needed. Second, there was no difference in the worst pain score within 24 h after the surgery between the two groups. On the other hand, the pain scores 6–24 h after surgery were significantly lower for those with TPVB than those without, even though we did not find a statistically significant difference in the pain scores at 0–3 h or at 3–6 h. In the patients who received TPVB, we did not perform cold or pin-prick tests, but the intraoperative remifentanil requirement was significantly less in the patients who received TPVB than in patients who did not. Hence, we can assume that TPVB was effective. Moreover, in the patients who received TPVB, fentanyl was not administered, as it was in those who did not receive TPVB (average dose, 1.55 ± 1.11 μg/kg), resulting in comparable pain scores 0–3 h after surgery between the two groups(Fig. 3). In addition, TPVB does not block the brachial plexus nerve; therefore, neither the pectoral nerve nor the long thoracic nerve would be blocked by TPVB. These regional deficiencies of analgesia are presumed to have affected the finding that there was no difference in the worst pain score within 24 h after the surgery between the two groups. Third, the patients recruited for this survey were heterogeneous and underwent different types of BCS. This heterogeneity might have affected the future occurrence of chronic pain. Fourth, the sample size was small.

### Conclusions

In conclusion, results of our study suggest that TPVB combined with general anesthesia may reduce the incidence of postoperative chronic pain for more than 1 year after BCS.

## Abbreviations

BCS: breast cancer surgery
BMI: body mass index
TPVB: thoracic paravertebral block
